# Evaluating Bacterial Nanocellulose Interfaces for Recording Surface Biopotentials from Plants

**DOI:** 10.3390/s24072335

**Published:** 2024-04-06

**Authors:** James Reynolds, Michael Wilkins, Devon Martin, Matthew Taggart, Kristina R. Rivera, Meral Tunc-Ozdemir, Thomas Rufty, Edgar Lobaton, Alper Bozkurt, Michael A. Daniele

**Affiliations:** 1 Department of Electrical and Computer Engineering, NC State University, Raleigh, NC 27606, USA; James.Reynolds@ncsu.edu (J.R.); Edgar.Lobaton@ncsu.edu (E.L.); aybozkur@ncsu.edu (A.B.); 2 Department of Crop and Soil Sciences, NC State University, Raleigh, NC 27695, USA; 3 Joint Department of Biomedical Engineering, NC State University and University of North Carolina, Chapel Hill, NC 27695, USA; 4 Department of Biology, University of North Carolina, Chapel Hill, NC 27599, USA

**Keywords:** nanocellulose, electrophysiology, plant, electrode

## Abstract

The study of plant electrophysiology offers promising techniques to track plant health and stress in vivo for both agricultural and environmental monitoring applications. Use of superficial electrodes on the plant body to record surface potentials may provide new phenotyping insights. Bacterial nanocellulose (BNC) is a flexible, optically translucent, and water-vapor-permeable material with low manufacturing costs, making it an ideal substrate for non-invasive and non-destructive plant electrodes. This work presents BNC electrodes with screen-printed carbon (graphite) ink-based conductive traces and pads. It investigates the potential of these electrodes for plant surface electrophysiology measurements in comparison to commercially available standard wet gel and needle electrodes. The electrochemically active surface area and impedance of the BNC electrodes varied based on the annealing temperature and time over the ranges of 50 °C to 90 °C and 5 to 60 min, respectively. The water vapor transfer rate and optical transmittance of the BNC substrate were measured to estimate the level of occlusion caused by these surface electrodes on the plant tissue. The total reduction in chlorophyll content under the electrodes was measured after the electrodes were placed on maize leaves for up to 300 h, showing that the BNC caused only a 16% reduction. Maize leaf transpiration was reduced by only 20% under the BNC electrodes after 72 h compared to a 60% reduction under wet gel electrodes in 48 h. On three different model plants, BNC–carbon ink surface electrodes and standard invasive needle electrodes were shown to have a comparable signal quality, with a correlation coefficient of >0.9, when measuring surface biopotentials induced by acute environmental stressors. These are strong indications of the superior performance of the BNC substrate with screen-printed graphite ink as an electrode material for plant surface biopotential recordings.

## 1. Introduction

Darwin and Burdon-Sanderson (1873) first discovered electrical activity in the Venus Flytrap by measuring the electrical current in the tissue when the plant was externally manipulated via feeding, touching, or cutting [1,2]. Since then, scientists have studied such potentials in a wide variety of plants ranging from tomatoes [3] to trees [4,5,6]. Furthermore, advances in statistical analysis and electrophysiological equipment allowed for the estimation of environmental stimuli based on the measured electrical signal [7]. The origin of this electrical activity in response to environmental stimuli is the internal, ionic-conduction-based long-distance signaling observed in cellular networks of the plant. This signaling utilizes reactive oxide species and ions via the plasmodesmata and the vascular system [8]. Being able to sense this activity can potentially provide a real-time notification of a plant’s electrophysiological response to an environmental condition. It has emerged as a potential phenotyping methodology due to recent improvements in the sensitivity and specificity of plant electrophysiology measurements [9,10].

Interfacing with plant electrophysiology using long-term, continuous measurements could lead to the real-time monitoring of plant health and stress responses, allowing for improved crop management, optimized sustainability, and even new sensing modalities that use the plant to detect environmental changes. To achieve this, there is a need for more effective and practical electrodes. Wet-gel-based electrodes, the current gold standard for human surface potential recordings such as electrocardiography, are ill suited for use with plants. Plant tissue is irreversibly damaged by the salt within the conductive hydrogel, which penetrates the tissue in a matter of hours [11,12,13,14]. This limits the experimental window to a short amount of time and can also interfere with the plant’s natural electrical behavior. Dry electrodes for use with plants typically take the form of metal clips or wires for surface measurements, which can block stomata and photosynthesis [15,16]. Invasive needle electrodes inserted directly into tissue could cause tissue damage and sometimes result in tissue encapsulation. More recently, aphid-based electrodes have been developed to improve the efficiency, but they introduce variability into the data and require intensive experimental labor [17]. Wet gel and needle electrodes are the only options that are commercially available and can be used for side-by-side benchmarking on the same experimental set up.

Screen-printed dry electrodes provide a potential alternative with many advantages over metal clip or wet gel electrodes for surface electrophysiology measurements. The ideal electrode substrate would be thin, light, vapor permeable, and optically transparent, particularly in the visible spectrum. In this study, we investigate nanocellulose as a potential substrate material. Cellulose is a glucose-based long chain polysaccharide that is one of the most abundant organic polymers in nature. It can be sourced from wood or algae, but this typically contains a mix of cellulose, hemicellulose, and lignin. Bacterial nanocellulose (BNC) is a pure form of cellulose produced as a defensive biofilm by various strains of bacteria. The high aspect ratio of BNC fibrils creates a unique mesh network when dried, yielding a material that is thin, light, strong, optically translucent, and vapor permeable [18,19]. BNC water vapor permeability is driven by adsorption from polar groups in the cellulose chain and the porosity of the matrix structure. Cellulose chains swell from adsorbed water vapor, shifting the BNC matrix structure and increasing porosity; thus, the water vapor permeability increases with relative humidity [20,21,22]. Dried BNC pellicles, with a structure like paper, fit well in existing screen printing workflows and thus can be patterned with conductive ink. We have chosen carbon ink for this study because it is flexible and more biocompatible than alternative options.

This work is an exploration of the performance of BNC electrodes with screen-printed carbon ink pads and traces for long-term monitoring of plant surface electrophysiology. First, we present our fabrication methods for generating BNC electrodes for plant surface measurements. Then, we present in vitro and in vivo characterizations of these electrodes for solar irradiance, water vapor permeability, stomatal conductance, relative chlorophyll content, and transpiration rate as proxies that would affect the health of the leaf tissue under electrodes over time. Finally, we present, as a proof of concept, the acquisition of surface electrophysiology signals in vivo from three model plants, *Zea mays* (maize), *Arabidopsis thaliana*, and *Manihot esculenta* (cassava), as a response to externally applied acute stressors.

## 2. Materials and Methods

### 2.1. Culture of Bacterial Nanocellulose

**Figure 1:**
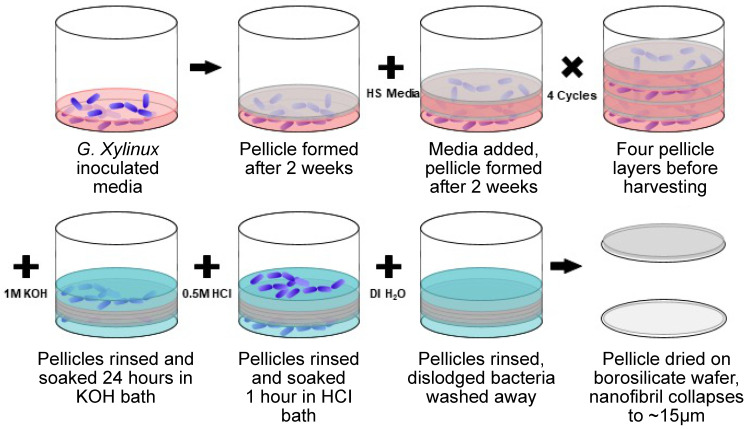
Bacterial nanocellulose production from *Glucanoacetobacter xylinus* in HS medium and subsequent purification with basic (KOH) and acidic (HCl) washes. Pellicles were separated and air dried to achieve the final films for electrode fabrication.

Bacterial nanocellulose pellicles were grown from a culture of *Gluconacetobacter xylinus* (ATCC 700178) using the methods shown in Figure 1 and as reported in our previous work [23]. Nanocellulose pellicles are produced by culture in a 30 °C incubator with a new layer being produced every two weeks. Pellicles were grown for two weeks before being covered with Hestrin and Schramm (HS) media. After four layers were grown, the pellicles were harvested. Thicker pellicles were produced by doubling the HS medium added between layers from 50 mL to 100 mL. Pellicles were harvested and rinsed in deionized (DI) water and bathed in 1 M KOH for 24 h. Following the base bath, pellicles were rinsed in 0.5 M HCl and DI water before finally drying on borosilicate wafers.

### 2.2. Fabrication of Bacterial Nanocellulose Electrodes

Screen-printed electrodes were fabricated onto approximately 10 µm-thick dry BNC pellicles via a custom screen-printing jig. Graphite ink (C2050106P7, Gwent Electronic Materials Ltd., Pontypool, UK) was deposited through a mounted and calendared 230 mesh stainless-steel trampoline screen (Sefar, Buffalo, NY, USA). Samples were then thermally annealed at a range of temperatures from 50 °C to 90 °C for 5 to 60 min, yielding conductive layers approximately 15 µm thick. Electrodes were uniformly separated using a laser cutter.

### 2.3. Characterization of Bacterial Nanocellulose Electrodes

Functional electrodes should be highly conductive and have a large effective surface area in order to be useful. To assess this, we specifically examined the electrical impedance and electrochemically active surface area (ECSA) of the carbon-ink-coated BNC electrodes. We used electrochemical impedance spectroscopy (EIS) to measure impedance and cyclic voltammetry (CV) to measure ECSA. Our procedures for CV and ECSA (detailed in [23]) can be summarized as follows: All electrochemical measurements were taken with a PalmSens4 potentiostat and impedance analyzer (PalmSens, Utrecht, The Netherlands) with all measurements made in triplicate (*n* = 3). CV was performed in an aqueous solution of 0.1 M KCl and 1 mM Hexaammineruthenium (III) chloride, [Ru(NH3)6]Cl3 (Sigma-Aldrich, St. Louis, MO, USA), using the fabricated carbon/graphite working electrodes, platinum wire counter electrode (Bioanalytical Systems, Inc., West Lafayette, IN, USA), and Ag/AgCl reference electrode (Pine Research Instrumentation, Inc., Durham, NC, USA). CV was performed at 5 different scan rates for 3 cycles each, 10, 25, 50, 100, and 250 mV·s^−1^, to determine the ECSA via the Randles–Ševčík equation:

$$I_{peak,forward}=nFAC\sqrt{nFvD/RT}$$

In this equation, *A* represents the effective area (in $m^{2}$), $I_{peak,forward}$ stands for the forward peak current (in Amps), and *v* is the scan rate (in V/s). All other terms—*n* (electrons transferred), *F* (Faraday constant), *C* (concentration), *D* (diffusion coefficient), and *R* (gas constant)—are constants or were controlled and can be treated as constants. This equation allows us to calculate the effective electrochemically active area from the peak current of the voltammogram.

To relate multiple BNC electrode manufacturing parameters to electrode properties, we created a simple second-order linear regression model in MATLAB. The linear regression models were fitted using MATLAB’s “fitlm” function. Since the testing space is meant to represent a reasonable range of the manufacturing parameters for BNC electrodes, we elected to use Sobol indices specifically as global sensitivity methods. With *D* being the total variance and $D_{i}$ representing the partial variances, the Sobol indices, $S_{i}$, and Sobol total indices, $S_{T_{i}}$, are:

$$S_{i}=\frac{D_{i}}{D}$$

$$S_{ij}=\frac{D_{ij}}{D}$$

$$\sum_{i=1}^{p}S_{i}+\sum_{1\leq i<j\leq p}S_{ij}=1$$

$$S_{T_{i}}=S_{i}+\sum_{j=1}^{p}S_{ij}$$

Two methods were employed for determining electrode impedance. First, the screen-printed electrodes were connected in a standard two-contact format for analysis via EIS. Next, we used two gold-plated printed circuit boards (PCBs) with a contact pad and a parallel RC circuit of known values (first PCB: 995 Ω and 15 pF; second PCB: 98.65 kΩ and 22.8 pF). A modified Randles model was used to determine the impedance and the values of R and C elements [23].

To test the water vapor permeability, BNC discs with thicknesses of 10 ± 4 μm were laser cut to be slightly larger than the 5 ${cm}^{2}$ opening of a 3D-printed evaporation well (Form 2, Formlabs, Somerville, MA, USA). Evaporation wells were filled with 10 mL of DI water. BNC discs were attached to the evaporation wells with gaskets to ensure evaporation only through the BNC. One set of BNC discs was soaked in a 5% pullulan solution for 5 min and allowed to dry before attaching to the evaporation wells. Pullulan was used as an adhesion promoter. Evaporation wells were placed in a humidity- and temperature-controlled (30% and 23 °C) environment for 72 h and weighed at intervals to determine the mass loss to evaporation. In addition to the BNC wells, uncovered wells and well covered with Parafilm (Bemis Company, Inc., Sheboygan Falls, WI, USA) were also placed in the chamber. This was conducted to evaluate the permeability of the electrodes, which is important for leaf transpiration through the electrodes.

Solar transmission measurements were used to assess if the electrodes block any light wavelengths critical for photosynthesis. For this, BNC discs with a thickness of 10 ± 4 μm were placed in a sample holder connected by a fiber optic cable to an ultraviolet–visible spectrometer (BLACK-Comet UV-VIS, StellarNet, Tampa, FL, USA). While the carbon ink forming the conductive layer blocks most of the light, it should be noted that it constitutes only a small portion of the electrode.

Three variants of electrodes were used for determining the effect of BNC electrodes on crop leaf chlorophyll production as a health indicator of the underlying plant tissue: a 500 ${mm}^{2}$ wet gel electrode (H124SG Covidien) normally used for electrocardiography or electromyography on humans, a 202 ${mm}^{2}$ BNC substrate with a 10 ${mm}^{2}$ carbon ink conductive layer (5% coverage), and a 202 ${mm}^{2}$ BNC substrate with no carbon conductive layer. The BNC electrodes were sized to be representative of the size needed for plant interfacing, but the wet gel electrodes were a standard size. The purpose of using the wet gel electrode was to demonstrate the importance of choosing the appropriate electrode for long-term measurements. All BNC substrates had a thickness of 10 ± 2 μm after drying down. They were applied to maize (*Zea mays* Agrisure® Viptera™ 3111) as a model plant. Plants were grown in a walk-in controlled-environment chamber at the North Carolina State University Phytotron. Seeds were directly sown in 6.1 L pots filled with a custom mix of 50% peat-lite, which itself is composed of 50% milled peat moss and 50% fine vermiculite, and 50% sand under 600 μmol/$m^{2}$ of photosynthetic fluorescent and incandescent light for 16 h daily light periods. The ambient chamber temperature was maintained at 28 °C during light periods and 22 °C during the dark. The relative humidity was approximately 50% for the duration of plant growth. Plants were watered as needed with DI water and a custom all-purpose nutrient solution was provided by the NC State University Phytotron [24].

In this paper, growth and leaf stages of maize are subdivided and designated numerically as “Vn”, where “n” represents the last leaf stage and “V” stands for vegetative stages. For example, V2 refers to the second leaf from the bottom with visible leaf collars. Electrodes were placed on the underside of maize leaves V5–V7 with the conductive material in contact with the leaf. BNC electrodes were soaked in a 2% pullulan (V-Labs, Inc., Covington, LA, USA) solution for 5 min before application to promote better adhesion. Electrodes (*n* = 3) were applied in a time-staggered fashion from 288 h to 12 h before measurement across 8 maize plants at growth stage V13. Electrodes were placed exclusively on leaves V5–V7. The chlorophyll content was measured using a Soil Plant Analysis Development (SPAD) Chlorophyll Meter SPAD-502 (Konica Minolta, Tokyo, Japan). Measurements were taken in triplicate at each electrode contact point after removing electrodes and at three reference points adjacent to the electrode site for each leaf used in the experiment.

A similar experimental setup was used to determine the stomatal conductance and transpiration rate after long-term exposure to BNC and pullulan. Larger 1050 ${mm}^{2}$ wet gel electrodes and 1250 ${mm}^{2}$ BNC substrate rounds were placed on multiple maize plants on leaves V5–V7. Maize plants for this experiment were in growth stage VT, which designates that tasseling has occurred. One set of BNC discs was soaked in 2% pullulan solution for 5 min before placement, and another set of BNC discs was soaked in DI water. To determine transpiration rate over a three-day period, locations were progressively measured after the removal of a BNC round with an LI-6800 Portable Photosynthesis System (LI-COR, Lincoln, NE, USA) equipped with a 2 ${cm}^{2}$ aperture. For comparison, a similar measurement was repeated on sites under wet gel electrodes for 48 h.

### 2.4. Recording of Plant Biopotentials and Electrophysiological Responses to Stressors

A proof-of-concept demonstration of the BNC electrodes’ biopotential acquisition capability was achieved by measuring the stress response of a maize plant to various external stressors. Simultaneous measurements were performed using conventional platinum–iridium needle electrodes (Technomed, Roseville, MN, USA) for comparison with the BNC electrodes, which had a 36 AWG enameled copper wire (Remington Industries, Johnsburg, IL, USA) attached to the carbon ink layer using a conductive epoxy (8331, MG Chemicals, Burlington, OR, Canada). The carbon ink layer was pressed against the plant tissue with the BNC, keeping it in contact with the plant. Needle electrodes provided a simple but effective means of creating an electrical connection [7,25,26]. The platinum–iridium needles have a lower impedance than their stainless-steel counterparts and thus can provide a better signal [27]. The amplifier for measuring the voltages (16-Channel Extracellular Differential AC Amplifier Model 3500, A-M Systems, Sequim, WA, USA) had a 200× gain, a 0.3 Hz high-pass filter, a 60 Hz notch filter, and a 100 Hz low-pass filter. An additional 15 Hz digital low-pass filter was applied during post-processing. The data were then analyzed by generating the correlation coefficient between the voltages recorded from the BNC electrodes and the needle electrodes as well as the percent difference between the maximum amplitudes of the two electrode types. Outliers more than two standard deviations from the mean were excluded. The measurement electrodes were located at the midpoint of leaves V8 and V9 on a single plant for an initial analysis and at V4 and V5 for a second broader analysis using four separate plants. The ground reference input was a needle in the substrate of the pot. Three different stressor stimuli (flaming, cutting, and cooling) were applied to a leaf to assess the electrophysiological response. The heat stressor involved holding a flame under the tip of the leaf for 3 s. The leaf cutting consisted of using electrically insulated scissors to slice the leaf in the middle. The cold response was the result of dropping 1 mL of ice-cooled water on the distal end of the leaf. When compared to flaming and cutting, cold water application has a shorter exposure time and a reversible physical effect that does not inflict permanent harm to the plant.

In parallel to maize, the BNC electrodes were also tested on both *Arabidopsis thaliana* and *Manihot esculenta* (Cassava) plants exposed to a subset of similar stressors. Following standard leaf numbering for *A. thaliana*, the nanocellulose electrode was attached on the underside of leaf 11, where the numbering is described in the literature [28]. The ground reference electrode was a stainless-steel needle inserted into the soil proximal to, but not in, the tap root. Similar to maize, q cuts using insulated scissors at the midpoint of the leaf and cold water droplets were used to generate an electrophysiological response. Cassava is an important food crop with a much longer maturation period compared to maize and *A. thaliana* [29]. This extended environmental exposure makes monitoring even more important, but it also means that the number of plants in a research setting is limited. As such, only cold water drops were used, rather than leaf cutting, for inducing stress in this model. The experiment consisted of six cassava plants with the nanocellulose electrodes attached to a central leaflet. A needle electrode in the soil near the stem served as the ground reference input to the amplifier channel. A pipette collected ice-cooled water droplets from an ice bath and released it over an alternate central leaflet.

These stressors are not necessarily representative of those expected in field conditions. They were chosen to evoke a significant long-distance signaling event in the plants in order to compare the performance of the presented nanocellulose electrodes with those of more conventional needle electrodes, as well as to demonstrate that the nanocellulose electrodes can be used on a variety of plants.

## 3. Results and Discussion

**Figure 2:**
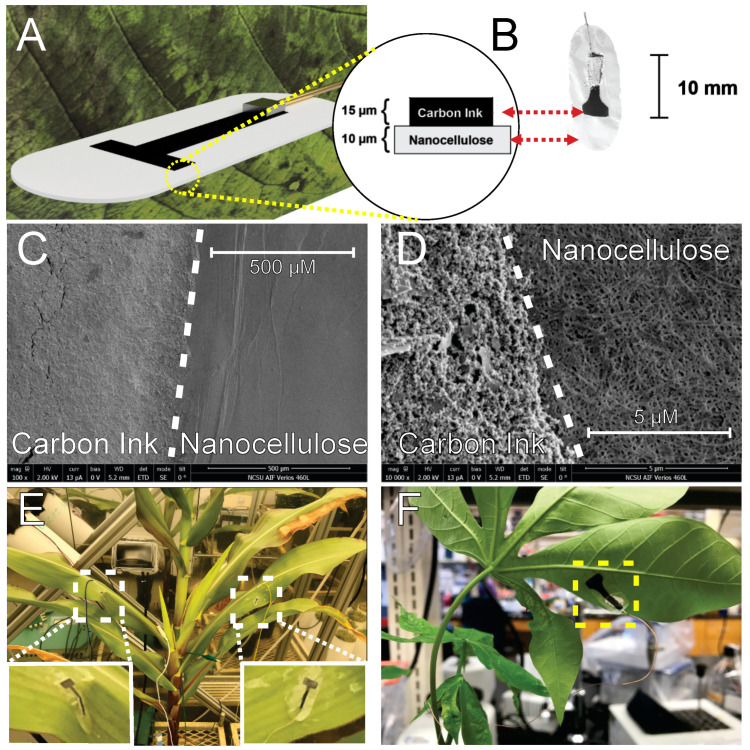
(**A**,**B**) The construction diagram of the BNC electrodes with attached wires for electrical connection. (**C**,**D**) Representative scanning electron micrographs of the carbon ink printed electrodes on nanocellulose films. (**E**,**F**) Photographs of as-applied electrodes on maize and cassava leaves, respectively.

These experimental efforts aimed to develop and characterize a new class of flexible electrodes that can record biopotentials on the surface of plant tissues with high fidelity and minimal deleterious effects to plant physiology. Nanocellulose-derived substrates were employed, since nanocellulose composites have been reported to exhibit good cytocompatibility, chemical stability, and processing robustness [30,31,32]. Specifically, BNC exhibits a high mechanical strength while maintaining flexibility and having unique water vapor and gas permeability properties thanks to its nanofibrillar structure [18,19,33]. Accordingly, after culture and harvest of BNC, we fabricated simple conductive pads on the BNC by screen printing conductive graphene paste on the nanocellulose. Screen printing of carbon-based conductors is an economical and high-throughput process that is popular for dry biopotential electrode manufacturing [34,35,36]. Figure 2 shows a representative illustration, photographs, and electron micrographs of the fabricated BNC electrodes. The scanning electron micrographs illustrate the contrasting particulate and fibrous morphology of the carbon ink and nanocellulose film.

### 3.1. Electrochemically Active Surface Area and Electrical Impedance

To characterize and optimize the BNC electrodes, we sought to correlate fabrication process conditions (i.e., BNC thickness, annealing time, and temperature) with critical performance characteristics (i.e., ECSA and impedance). From a manufacturing perspective, the most important parameters in electrode production are the annealing time, annealing temperature, and BNC thickness. The annealing time and temperature influence ink brittleness, while the BNC thickness impacts vapor permeability and the carbon ink penetration depth. Accordingly, each of these processing outcomes are important to the critical performance characteristics of any biopotential electrode, the electrical impedance and the ECSA, as these relate to charge transport at the interface.

To characterize the relationship between manufacturing conditions and the BNC electrode properties, we analyzed the controllable manufacturing conditions with the following input ranges: 5–60 min for time, 50–90 °C for temperature, and 10–20 μm for thickness. A total of 22 different input points were evaluated, each in triplicate (66 in total), with corresponding outputs consisting of the individual electrode impedances and ECSA.

**Figure 3:**
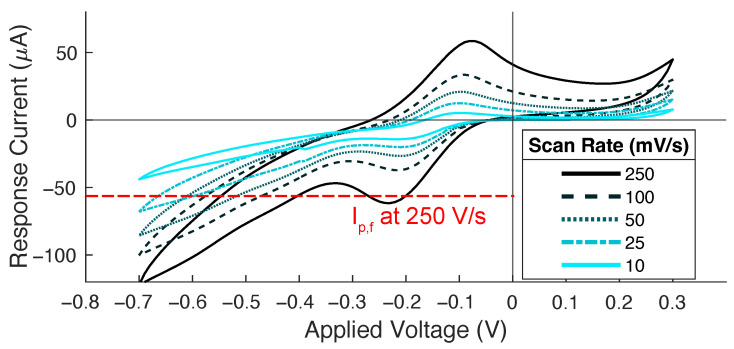
A representative cyclic voltammogram plot used to evaluate the electrochemically active surface area where the increased scan rates yield higher-magnitude patterns.

We used CV and EIS to evaluate the ECSA ($m^{2}$) and impedance (Ω), respectively, for each electrode. The peak of the cyclic voltammogram was taken as the maximal forward current (see Figure 3) to use in the Randles–Ševčík equation and to determine the corresponding area. The process was performed for both the 0.25 V/s and 0.10 V/s scan rates since these produced the most prominent peaks. The impedance was estimated from the EIS curves using circuit fitting in PSTrace (PalmSens BV, PSTrace5 version 5.9.4515) after fitting them to a simple resistance-only circuit.

We checked the correlations between the ECSA and impedance using a scatterplot matrix, which indicated that each of the outputs is approximately normally distributed, suggesting successful sampling. Typical values were measured to be $1.93\times 10^{-4}$ ± $4.62\times 10^{-5}$$m^{2}$ for the ECSA at 0.25 V/s, $1.77\times 10^{-4}$ ± $3.96\times 10^{-5}$$m^{2}$ for the ECSA at 0.10 V/s, and 473 ± 74 Ω as the impedance. The ECSA between 0.25 and 0.10 V/s scan rates showed a strong correlation (r = 0.95), indicating that only one of these would be sufficient for modeling. We used 0.25 V/s for its higher peak prominence. All other output pairs showed low or no correlation.

**Figure 4:**
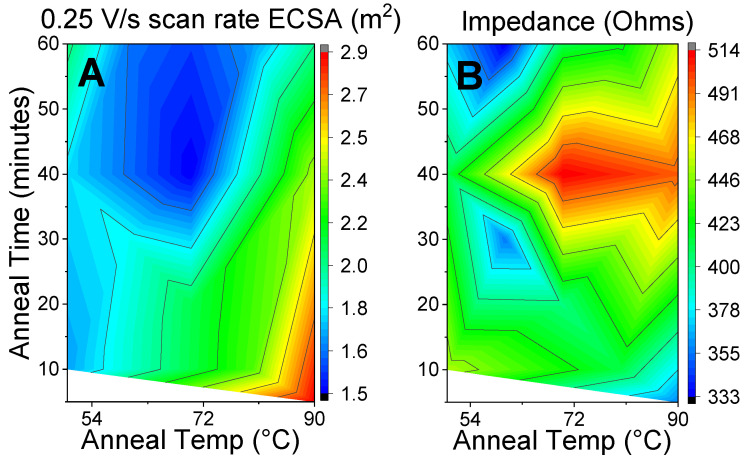
(**A**) The electrochemically active surface area and (**B**) the electrical impedance of electrodes as a function of the annealing temperature from 50 to 90 °C and annealing time from 10 to 60 min.

Simple one-to-one linear regressions were modeled to check for dependence on each individual input. The data distribution looked as expected. Generally, higher curing temperatures result in higher ECSAs and impedances. To a lesser degree, longer curing times yield lower ECSAs and impedances. Lastly, the BNC thickness had no effect on the ECSA but did increase impedance. Figure 4 shows the surface plots generated from the annealing time and temperature to the ECSA and impedance. Optimizing for a high ECSA and low impedance requires a high annealing temperature and a short duration of around 90 °C and 10 min, respectively.

**Figure 5:**
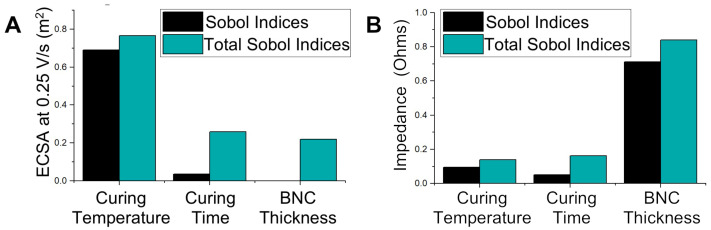
Sobol and total Sobol indices relating BNC electrode manufacturing parameters to (**A**) ECSA and (**B**) impedance. High indices indicate a strong relationship between the manufacturing parameter and electrode property. The curing temperature was a strong indicator of ECSA and thickness was strongly related to impedance in the linear regression model.

The Sobol indices provide a measure of the degree to which the variability of any input variable has an impact on any output parameter. The higher the Sobol index, the greater the dependency between the variables. Additionally, the first Sobol index relates the effects of just changing one variable, while the total Sobol index includes every pairwise and higher-order-wise effect from that one variable [37]. The first and total Sobol indices are shown in Figure 5. The curing temperature of the conductive graphene paste was the most significant effector of the ECSA. The curing time had little effect on either the ECSA or impedance; thus, the optimal configuration agrees with Figure 4: a high curing temperature for short times. While the nanocellulose thickness had a negligible effect on the ECSA, it significantly impacted the resultant impedance of the BNC electrodes. This could be due to a number of factors, including the surface roughness and BNC porosity, which would affect the conductive graphene ink thickness [38].

### 3.2. Water Vapor Permeation Rate and Plant Health Effects

**Figure 6:**
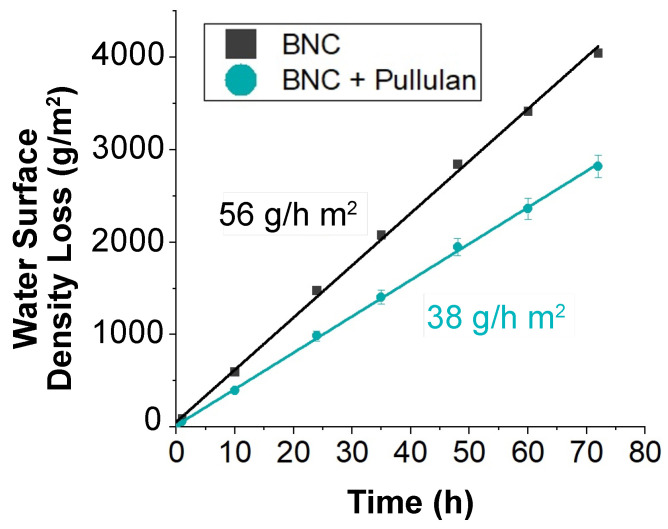
Water vapor permeation rate through bare bacterial nanocellulose and through bacterial nanocellulose soaked in a 5% pullulan solution.

As a part of the respiration process, plants constantly exchange gases through their leaves. Blocking this transpiration results in cell death. The water vapor transmission rate can be used to assess how transpiration is affected. The water vapor transmission for the BNC substrates was measured at an average evaporation rate of 56 g/hr $m^{2}$, which was an improvement over other adhesive substrates (Table 1). When combined with the adhesion-promoting pullulan, the evaporation rate was reduced to 38 g/hr $m^{2}$, a 32% reduction, due to pullulan (Figure 6). This evaporation rate is between the rate for a commonly used thermoplastic substrate Parafilm™ (Bemis Company, Inc, Neenah, WI, USA), which was effectively 0 g/hr $m^{2}$ over this time scale, and an open container with a rate of 340 g/hr $m^{2}$.

**Figure 7:**
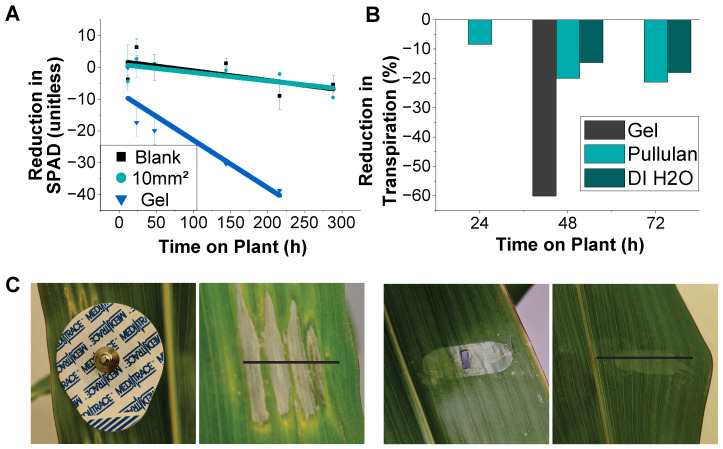
(**A**) Reduction in chlorophyll content caused by electrodes on the leaf surface with reference to an adjacent, uncovered leaf surface. (**B**) Relative reduction in transpiration over time due to the electrode substrate and adhesion method. (**C**) Representative photographs of plant tissue damage three days after the application of a standard wet gel electrode and the reported nanocellulose interface.

The relative chlorophyll contents and transpiration rates were used as proxies for determining the health of the leaf tissue under electrodes over time. As the plant tissue dies, the amount of chlorophyll decreases. The reduction in chlorophyll over 300 h was similar between blank BNC and BNC with a graphene conductive layer with an area of 10 ${mm}^{2}$, as shown in Figure 7A. The average reduction in chlorophyll was −3.2 ± 1.7 for the blank BNC and −7.6 ± 0.49 with the ink, demonstrating that the effect of the conductive ink was minimal. As mentioned earlier, gel electrodes have been used in the literature for measuring electrical signaling in plants, but are not suitable for long-term experiments, as they have a more significant impact on the chlorophyll content and transpiration than BNC films. The measurements of the relative reduction in transpiration rates in the area under these electrodes can be found in Figure 7B. BNC electrodes reduced transpiration by ~20% over 72 h, but gel electrodes reduced transpiration by 60% in just 48 h. The pullulan-adhered BNC electrodes exhibited a slightly higher transpiration reduction than BNC electrodes transferred with DI water, which could be attributed, at least partially, to the reduction in water vapor permeability of BNC substrates when soaked in pullulan.

### 3.3. Solar Light Transmission

**Figure 8:**
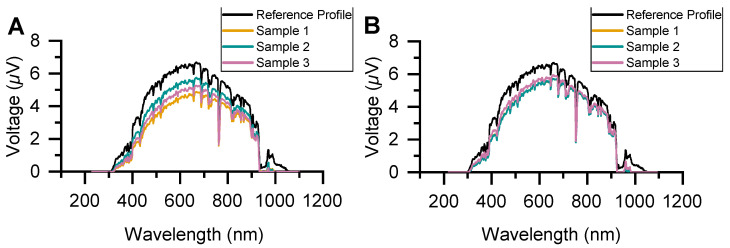
Solar transmission spectrum for (**A**) dry and (**B**) wet nanocellulose films. Dry samples exhibit a reduction in transmission with increasing film thickness. All spectra are an average of *n* ≥ 3 measurements. Reference profiles are for full sun exposure.

The plant tissue damage is also directly correlated with any occlusion from solar irradiance. Accordingly, we characterized the solar light transmission of different nanocellulose films, given that nanocellulose forms the majority of the electrode and that its outline was visible on the plants. Solar light transmission characterization was conducted outdoors using a Black Comet UV-Vis Spectrometer (StellarNet Inc.). Measurements were conducted around noon on sunny days. Figure 8 exhibits the average spectral transmission measurements of three identical nanocellulose disk samples when dry and wet. The nanocellulose films exhibit good transparency across the solar spectrum, with any reduction directly correlated with the nanocellulose film thickness. This can be attributed to surface scattering, which was also supported by the comparison between dry and wet films [41,42].

### 3.4. Biopotential Recordings and Functional Assessment

Having optimized the design of the BNC electrodes and demonstrated their minimal effect on plant health, the next step was to evaluate their effectiveness at measuring electrical activity in plants. Compared to needle electrodes (or other types that pierce the plant), surface electrodes can potentially have a higher electrical impedance due to the electrically insulating nature of the outer tissue layers such as the waxy cuticle. By comparing the BNC electrodes against needle electrodes, we were able to evaluate how well our electrodes serve as a non-invasive alternative.

**Figure 9:**
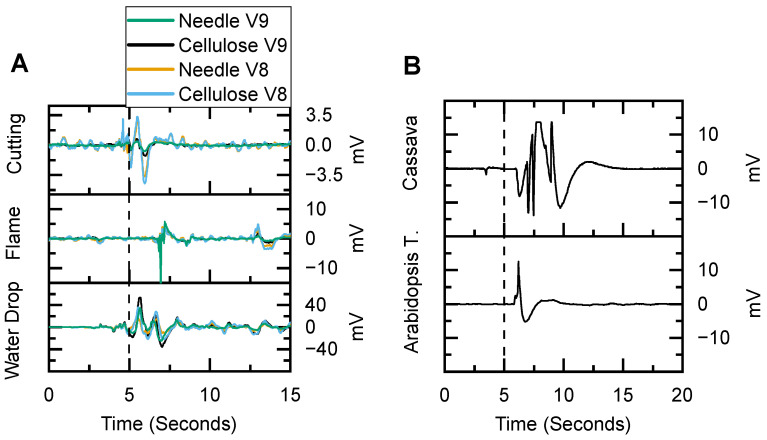
(**A**) Representative maize action potentials caused by acute stressors and measured with BNC and needle electrodes. (**B**) Representative electrical signaling from cassava and *Arabidopsis thaliana* plants acquired with BNC electrodes for proof-of-concept.

We measured the electrical activity across several plant species and multiple plants to show the electrode’s consistency. The performance of BNC electrodes for measuring surface potentials was comparable to that of the platinum–iridium needle electrodes inserted into plant tissue. The BNC electrodes were also effective across multiple species. Representative surface potentials measured using BNC electrodes in maize, *A. thaliana*, and cassava are shown in Figure 9, with the dashed line indicating when a stressor was introduced.

In experiments performed on a maize plant with both BNC and needle electrodes, the responses were visually similar (Figure 9A). For quantitative comparison, we used the correlation coefficient, where a value of 1 indicates a perfect fit and a value of 0 indicates no correlation, to objectively evaluate how well the signal from the BNC electrodes matched that of the needle electrodes. The BNC and needle electrodes had an average correlation coefficient of 0.92 ± 0.12 (*n* = 14) when the electrical response to stimuli was measured on leaves V8 and V9.

**Figure 10:**
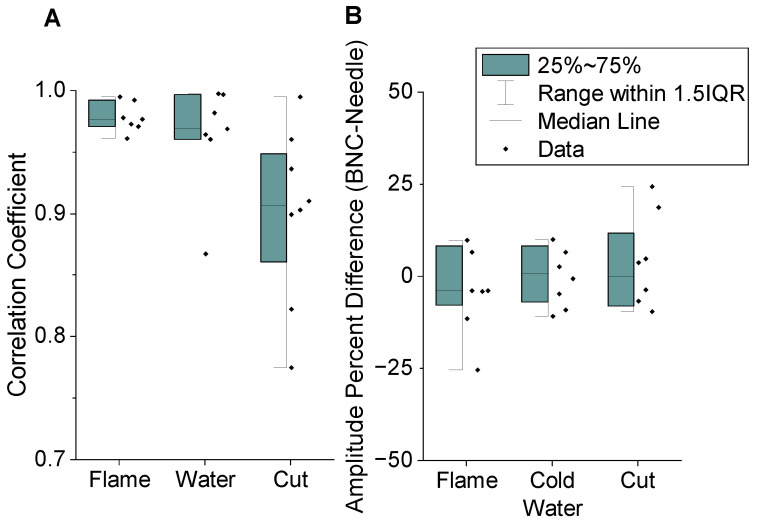
Comparison of signals acquired by BNC electrodes and needle electrodes as measured from V8 and V9 when acute stress was applied to V11: (**A**) correlation coefficient between BNC and needle electrodes and (**B**) percentage difference in amplitudes with extreme outliers not shown.

Based on this initial finding on a representative maize plant, the BNC and needle electrodes on two individual leaves were compared across four younger maize plants (detailed in Figure 10A). The average correlation coefficient between the BNC and needle electrodes was 0.94 ± 0.06 (Pearson’s, *n* = 22, *p* < 0.001). As for the applied stressors, the cutting stimulus resulted in the most variation. This may be indicative of this specific stimulus having different physiological effects that could affect a broader area and result in some minor differences to be investigated in the future. It should be noted that these outcomes serve as proof-of-concept demonstrators. Their detailed analyses require a deeper investigation, which is beyond the scope of this paper and reserved for a future study. Regardless, the overall correlation between the electrode types was very good, indicating that the presented BNC electrode design measures similar electrical patterns to those of the needle electrodes.

While the correlation coefficient compares how similar the electrical waveforms are, it does not compare magnitude, which determines how easy it is to measure the plant signals. A lower-magnitude signal requires more amplification and is more susceptible to noise. For the first maize plant, the average (*n* = 16) amplitude measured with the BNC electrode was 24 ± 48% larger than the needle electrode. The results, however, were not significantly different (Welch’s *t*-test, t (1.25) = 23.9, *p* = 0.222). The set of four plants had a similar range, with the signal amplitude from the BNC electrode being 5 ± 38% larger on average (*n* = 24) than that of the needle electrode. This difference, however, was not significant (Welch’s *t*-test, t (0.391) = 41.2, *p* = 0.698). Overall, this showed that the electrical signal recordings taken using BNC electrodes were generally very similar to those of the needle electrodes and can be considered as a suitable alternative.

We also tested these electrodes on *A. thaliana*, as one of the most commonly used models in plant science studies, to observe its electrical activity and response. In each of the 17 plants, a surface potential was clearly observed. These surface potentials had an average amplitude of 10.41 ± 1.68 mV and a transmission delay of 4.13 ± 0.89 s. For the six cassava plants, the surface potential amplitudes averaged 6.72 ± 4.29 mV. These measurements are in the same range as similar stress-induction experiments that had a propagation rate of over 1 mm per second and an amplitude of 5 to 100 mV [43,44], demonstrating that relevant results can be obtained using BNC electrodes.

## 4. Conclusions and Future Work

Further study of the surface electrophysiology of plants would enable new and effective phenotyping tools to understand plant stress responses and perception. BNC is a promising substrate material to adhere to the surface of the plants while still allowing for sunlight transmission to the chlorophyll and for vapor permeation. Screen printing of carbon ink on BNC facilitates a simple microfabrication process to generate electrically conductive pads and interconnecting traces. In this study, we presented a process flow to fabricate such flexible and breathable surface electrodes and shared our preliminary results when we adhered these to the surface of three model plants. Our analysis demonstrated that the BNC electrodes were superior to commonly used wet gel electrodes, reducing the chlorophyll content by just 16% and transpiration by only 20% compared to a 60% reduction under wet gel electrodes. Our preliminary in vivo assessment reveals that the BNC electrodes are just as effective at detecting surface biopotentials as invasive needle electrodes, where the recorded signal amplitudes were comparable and the waveforms were almost identical. The BNC electrodes were able to achieve these results without causing direct tissue damage due to insertion of needles or indirect damage due to the salt contained in conductive gels. The next stages of this research will focus on the plant science analysis and involve the use of BNC electrodes to understand, deconstruct, and isolate plants’ stress responses in the presence of various environmental stimuli that represent field conditions.

## Figures and Tables

**Table 1:** A comparison of the thickness and evaporation rates between similar adhesive materials.

Substrate	Thickness (μm)	Evaporation Rate (g/$dm^{2}$)	Reference
Bacterial Cellulose Composite	300	500	[39]
Tegaderm^TM^	50	283	[40]
Bacterial Nanocellulose	15	1344	This Work

## Data Availability

Data can be made available upon request by contacting the author.

## Abbreviations

The following abbreviations are used in this manuscript:

BNC	Bacterial Nanocellulose
DI	Deionized
SPAD	Soil Plant Analysis Development
ECSA	Electrochemically Active Surface Area
EIS	Electrochemical Impedance Spectroscopy
CV	Cyclic Voltammetry
PCB	Printed Circuit Board
